# Potential role of 25(OH)D insufficiency in the dysfunction of glycolipid metabolism and cognitive impairment in patients with T2DM

**DOI:** 10.3389/fendo.2022.1068199

**Published:** 2022-12-23

**Authors:** Hui-min Sun, Yue Yu, Xin-ran Gao, Ya-dong Wei, Chuan-zong Qi, Meng-die Ma, Dan-dan Xu, Ya-yun Xu, Jin-fang Ge

**Affiliations:** ^1^ School of Pharmacy, Anhui Medical University, Hefei, China; ^2^ The Key Laboratory of Anti-inflammatory and Immune Medicine, Ministry of Education, Anhui Medical University, Hefei, China; ^3^ Anhui Provincial laboratory of inflammatory and immunity disease, Anhui Institute of Innovative Drugs, Hefei, China; ^4^ Department of Pharmacy, North district of The First Affiliated Hospital of Anhui Medical University, Hefei, China; ^5^ School of Public Health, Anhui Medical University, Hefei, China

**Keywords:** T2DM, HbA1c, 25(OH)D, BRIEF-A, IL-6, CRP, sTREM1

## Abstract

**Purpose:**

To investigate the changes of plasma 25(OH)D levels in type 2 diabetes mellitus (T2DM) patients and explore its role in the dysfunction of glucose and lipid metabolism and cognition.

**Methods:**

One hundred and thirty-two T2DM patients were enrolled and the demographic and clinical data were collected. The plasma concentration of 25(OH)D was detected and the patients were divided into two groups including a Vitamin D insufficient (VDI) group and a normal VD group according to the clinical diagnostic criterial of VDI with the plasma 25(OH)D level less than 29 ng/mL. The glycolipid metabolic and routine blood biochemical indices were detected, the plasma concentrations of C-reactive protein (CRP), interleukin-6 (IL-6), soluble myeloid soluble trigger receptor 1 (sTREM1) were measured. The cognitive function was assessed using the Behavior Rating Inventory of Executive Function-Adult Version (BRIEF-A). The depressive symptomatology was assessed using the Center for Epidemiological Survey Depression Scale (CES-D). Sleep quality was assessed using the Pittsburgh sleep quality index (PSQI).

**Results:**

There were 70 T2DM patients with VDI (70/132, 53.03%) in this study. The plasma concentrations of glycated hemoglobin (HbA1c), fasting plasma glucose (FPG), postprandial blood glucose (PBG), IL-6, and sTREM1 were remarkably increased in T2DM patients with VDI as compared with that with the normal VD, accompanied with an elevated BRIEF-A scores. There was no significant difference between groups with regard to the indices of blood lipid, liver function, and scores in CES-D and PSQI. Moreover, results of Pearson correlation test showed that the plasma 25(OH)D levels were negatively correlated with HbA1c, FPG, PBG, CRP, IL-6, sTREM1, CES-D sum scores, and PSQI sum scores, but positively correlated with the plasma levels of Serum creatinine (Scr). Furthermore, result of Receiver Operating Characteristic (ROC) curve analysis showed a predictive role of VDI levels in discriminating T2DM patients with higher cognitive impairments, with the sensitivity and specificity being 62.12% and 62.12%, respectively.

**Conclusion:**

VDI is harmful for T2DM patients with a significant relation with the hyperglycosemia and cognitive dysfunction.

## Introduction

Type 2 diabetes mellitus (T2DM) is a common metabolic disorder characterized by the persistent hyperglycemia and insulin resistance. According to the report from the International Diabetes Federation (IDF), there are 537 million adult patients with diabetes mellitus, that is about 1 in 10 adults globally (1). In China, 140 million adults are diagnosed as T2DM in 2021 and the number is estimated to 170 million in 2045 (1). Due to T2DM patients are often accompanied by the chronic damage and dysfunction of blood vessels, nerves and brain (2, 3), most patients with diabetes have impaired cognitive function (4) or Alzheimer’s Disease (AD). Results of epidemiological perspectives indicated that the relative risk for dementia was 1.51 in people with T2DM compared with those without, and 25~36% of the patients have mild cognitive impairment (5). Unfortunately, there are no specific measures for preventing or treating the symptoms (6), and traditional hypoglycemic drugs are always low targeted. Thus, it is important to investigate the potential mechanism of T2DM-related cognitive dysfunction and explore the possible biomarkers for effective diagnosis and treatment.

The multifactorial process of cognitive dysfunction in T2DM is not yet completely understood, however, classical factors, such as oxidative stress and systemic inflammatory responses, may contribute to the pathogenesis of diabetes-related cognitive dysfunction (7, 8). It has been reported that cognitive impairment in T2DM rats can be improved by reducing inflammatory response (9). Among them, inflammatory cytokines IL-6 and CRP were significantly increased in T2DM patients with cognitive impairment (10). This may be due to the overproduction of pro-inflammatory factors in diabetes, which leads to changes in various neurotransmitters and damage to neurons (11). As a family of activating receptors, TREMs participate in the regulation of inflammatory response (12). The disruption of TREMs could exert an important influence on homeostatic activity (13), resulting in different outcomes in different diseases (14). Polymorphism and changed expression of TREM1 and TREM2 have been demonstrated to involve in the pathogenesis of neuropsychiatric diseases including AD (15). Apart from an imbalanced protein expression of TREM1 and TREM2 in the hippocampus of depression (16) or AD (17) rat model, results of our previous study have shown a turbulence of TREM1 and TREM2 abundance in T2DM patients (18).

Besides inflammation, depression and sleep disturbances have also been reported to play an important role in the pathogenesis of T2DM-related cognitive dysfunction. T2DM patients with depression had higher level of hyperglycemia and cognitive impairment than T2DM patients (19). Both depression and diabetes are accompanied by insulin resistance, which is the main reason for the high co-morbidity of the two diseases (20). And insulin resistance often exacerbates abnormalities in brain metabolism, showing cognitive impairment, which makes diabetes often accompanied by depression and cognitive impairment (21). In addition, it has been reported that T2DM mice with obstructive sleep apnea can damage neurons and lead to cognitive dysfunction (22). Other studies have shown that T2DM patients with cognitive impairment have poorer sleep quality, which is also associated with neuropathic pain, anxiety and depression, while patients taking sleep enhancing drugs have improved cognition (23, 24).

Vitamin D is a steroid hormone with versatile physiological activities (25). Recently, much attention has been paid to the effect of 25(OH)D in T2DM and its associated diseases (26, 27). Results from human studies have demonstrated that low 25(OH)D levels are associated with an increased risk of T2DM, including gestational diabetes mellitus (GDM) (28). Consistently, supplementation of 25(OH)D could not only prevent and treat GDM (29), but also contributed to blood glucose control and lipid balance in T2DM patients (30). Moreover, the “neuroprotective” effect of 25(OH)D has been demonstrated (31). It has been reported that lower 25(OH)D levels could accelerate the impaired hippocampal neurogenesis and cognitive decline in elder *via* promoting inflammatory response (32), and Vitamin D supplementation can improve the physical and cognitive performance (33). Similar conclusions were also obtained in animal models, in which the brain inflammation of T2DM rats supplemented with Vitamin D was reduced and the learning and cognitive abilities were significantly improved (34). These results indicated that 25(OH)D levels might be a diabetes risk modifier and linked to the cognitive impairment. However, its association with risk factors for the cognitive impairment remains to be further studied.

The aim of the present study is to investigate the role of VDI in the dysfunction of glucolipid metabolism and cognitive impairment in T2DM patients, especially the potential linking effect of cognitive function and its risk factors. Accordingly, T2DM patients were enrolled, the plasm concentration of activated 25(OH)D were measured, the parameters of inflammation and glucolipid metabolism were detected, and the cognitive ability, depression and sleep status were evaluated in the present study.

## Materials and methods

### Study context and target population

All patients were continuously recruited and diagnosed as T2DM by the Department of Endocrinology, The North district of the first Affiliated Hospital of Anhui Medical University from April 2017 to April 2018, including 132 patients. All diabetic subjects met the Chinese guidelines for the prevention and treatment of T2DM.

### Inclusion and exclusion criteria

The inclusion criteria include: 1) T2DM diagnosed according to the 1999 diagnostic criteria of the World Health Organization; 2) aging≥18 at the time of recruitment; 3) without any drug treatment; 4) without language communication disorders.

The exclusion criteria include: 1) suffering from acute diabetic complications, such as diabetic ketoacidosis and severe hypoglycemia coma; 2) suffering from active depression, epilepsy, head injury, or other mental or nervous system diseases; 3) severe systemic disease, such as thyroid disease, serious infection, and anemia; 4) diagnosed with alcohol or other substance-dependent diseases (18).

The research plan has been approved by the Ethics Committee of Anhui Medical University (No.20170245). All subjects provided written informed consent in accordance with the Declaration of Helsinki.

### Measurement of 25(OH)D levels and the metabolic parameters

The height and weight of all subjects were measured, based on which the BMI was calculated. In order to avoid the impact of daily energy intake, all patients were given the same food provided by the canteen of the North district of the first Affiliated Hospital of Anhui Medical University. Blood samples were collected from the participants’ cubital veins from 8:00 a.m. to 9:00 a.m. after fasting for 10 hours. The blood was centrifugated at 4°C with 2500 rpm for 20 minutes, and the plasma samples were collected and stored at 80°C until detection. The plasma concentrations of 25(OH)D, CRP, IL-6, sTREM1, and sTREM2 were determined by ELISA Kit according to the manufacturer’s instructions (25(OH)D, CRP, and IL-6: Huamei Bio, Wuhan, China; and sTREM1, sTREM2 and insulin: Meilian Bio, Shanghai, China). The plasma levels of Total plasma cholesterol (TC), triglyceride (TG), high-density lipoprotein (HDL), low-density lipoprotein cholesterol (LDL), alanine aminotransferase (ALT), aspartate transaminase (AST), total bilirubin (TBIL), direct bilirubin (DBIL), indirect bilirubin (IBIL), serum creatinine (SCR), blood urea nitrogen (BUN), fasting blood glucose (FPG), postprandial blood glucose (PBG), and glycated hemoglobin (HbA1c) were determined in the Clinical Laboratory Department of the North district of the first Affiliated Hospital of Anhui Medical University.

Clinically, 25(OH)D< 29 ng/mL is defined as VDI, among which the level less than 20 ng/mL is taken as VDD (35). Accordingly, the patients were divided into VDI group and normal VD group.

According to the 2010 American Diabetes Association Diabetes Management Guidelines, plasma HbA1c > 7% was associated with an increased risk of diabetes complications, especially microvascular complications. Therefore, the patients were divided into two groups if necessary, according to the 7% boundary, that is, a HbA1c ≤ 7% group and a HbA1c > 7% group.

### Cognitive assessment neuropsychiatric

The cognitive function was assessed using the adult version of the executive functional Behavior evaluation table (BRIEF-A). BRIEF-A includes two comprehensive scores. Specifically, inhibit, shift, emotional control, and self-monitor scale are used to calculate the behavior adjustment index (BRI). Initiate, working memory, plan/organization, task monitor, and organization of materials are used to calculate metacognitive index (MI).

### Depressive symptoms

The depressive symptoms measured using the Center for Epidemiologic Studies Depression Scale (CES‐D). CES‐D was mainly used for screening out subjects with depressive symptoms in epidemiological investigations, and also used as a clinical measure to assess the severity of depressive symptoms. The scale contains 20 items and measures the frequency and severity of depressive symptoms. The total score ranged from 0 to 60, with higher scores indicating higher levels of depression.

### Sleep quality

Sleep quality was assessed using the Pittsburgh sleep quality index (PSQI), which is a scale used to assess subjective sleep disturbances in patients over the past month. The scale contains 19 self-rating items, which can be divided into seven components, and has a total score of 21 points, with higher scores indicating worse sleep quality.

### Statistical analysis

The data is input into EpiData version 3.1 and analyzed using SPSS version 17.0 statistical software. Kolmogorov-Smirnov test was used to test the normal distribution of continuous variables. Student’s *t* test or Chi-square test was used for comparison between groups. Correlation analysis was carried out using the Pearson test. Receiver Operating Characteristic (ROC) curve analysis was used to determine the cutoff values of area under curve (AUC) and 25(OH)D, and to identify T2DM patients with HbA1c< 7% and BRIEF-A total score below 50%. Data were analyzed with descriptive statistics $\bar{x}$ (SD or percentage), and *P<*0.05 was considered statistically significant.

## Results

### Demographic characteristics of participants

We recruited 228 patients and screened 132 patients with T2DM for analysis, the selection process for the data is shown in **Figure S1**. Detailed information regarding characteristics of participants in the total sample as well as the two analyses subsamples is given in **Table 1**. According to clinical diagnosis criteria of VDI, there are 70 T2DM patients with VDI (25(OH)D< 29 ng/ml, 70/132, 53.03%) and 62 with normal VD level (25(OH)D ≥ 29 ng/ml, 62/132, 46.97%). There were no significant differences of age, gender, BMI, WHR, drinking or smoking status, and diabetes history and complications between T2DM patients with VDI and normal VD groups.

**Table 1:** Information on demographic and medical history in the total sample as well as the VDI and normal VD subsamples.

Variables	Total Sample (n=132)	VDI Sample (n=70)	Normal VD Sample (n=62)	*t* or *χ^2^* value	*P* value
**Age,year**	57.47±1.08	56.27±1.43	58.82±1.62	1.19	0.24
Sex, n					
** Females**	62 (46.97%)	35 (50.00%)	27 (43.55%)	0.55	0.46
** Males**	70 (53.03%)	35 (50.00%)	35 (56.45%)		
**BMI (kg/m^2^)**	24.20±0.33	24.58±0.39	23.61±0.47	1.6	0.11
** WHR**	0.92±0.01	0.92±0.01	0.91±0.01	1.08	0.28
Smoking status					
** Smoker**	88 (66.67%)	47 (67.14%)	41 (66.13%)	0.02	0.9
** Non-smoker**	44 (33.33%)	23 (32.86%)	21 (33.87%)		
Drinking status					
** drinker**	48 (36.36%)	26 (37.14%)	22 (35.48%)	0.04	0.84
** Non-drinker**	84 (63.64%)	44 (62.86%)	40 (64.52%)		
Diabetes duration, n					
** <5 years**	63 (47.73%)	37 (52.86%)	26 (41.93%)		
** 5-10 years**	43 (32.58%)	20 (28.57%)	23 (37.10%)	1.22	0.54
** >10 years**	26 (19.70%)	13 (18.57%)	13 (20.97%)		
Family history of diabetes					
** Have**	58 (43.94%)	33 (47.14%)	25 (40.32%)	0.62	0.43
** one**	74 (56.06%)	37 (52.86%)	37 (59.68%)		
Diabetes peripheral neuropathy					
** Have**	69 (52.27%)	34 (48.57%)	35 (56.45%)	0.82	0.37
** None**	63 (47.73%)	36 (51.43%)	27 (43.55%)		
Diabetic retinopathy					
** Have**	24 (18.18%)	10 (14.29%)	14 (22.58%)	1.52	0.22
** None**	108 (81.82%)	60 (85.7%)	48 (77.58%)		
Diabetic nephropathy					
** Have**	13 (9.85%)	9 (12.86%)	4 (6.45%)	0.88	0.35
** None**	119 (90.15%)	61 (87.12%)	58 (93.55%)		
Diabetic peripheral vasculopathy					
** Have**	90 (68.18%)	47 (67.14%)	43 (69.35%)	0.07	0.79
** None**	42 (31.82%)	23 (32.86%)	19 (30.65%)		
Diabetes combining hypertension					
** Have**	56 (42.42%)	29 (41.43%)	27 (43.55%)	0.06	0.81
** None**	76 (57.58%)	41 (58.57%)	35 (56.45%)		

### Turmoil changes of plasma levels of HbA1c and 25(OH)D in T2DM patients and their close correlation

**Figure 1:**
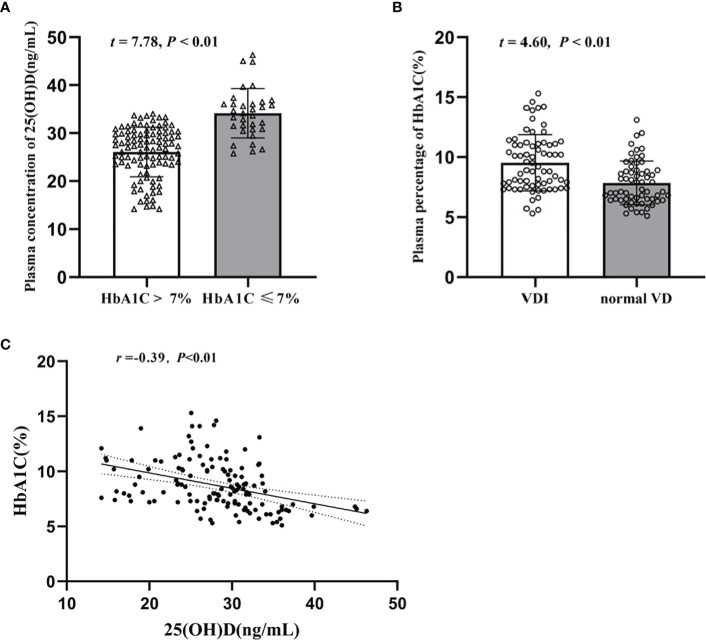
Turmoil changes of plasma levels of HbA1c and 25(OH)D in T2DM patients and their close correlation. **(A)** Plasma concentration of 25(OH)D (ng/mL) in HbA1c>7% and HbA1c ≤ 7%; **(B)** Plasma percentage of HbA1c (%) in VDI group and normal VD group; **(C)** Correlation between plasma 25(OH)D and plasma HbA1c levels.

As shown in **Figure 1A**, the plasma 25(OH)D level of T2DM patients in HbA1c > 7% group was significantly lower than that of the HbA1c ≤ 7% group [(34.16 ± 5.16) ng/ml V.S. (26.07 ± 5.18) ng/ml, *t* = 7.78, *P*<0.01]. In turn, as shown in **Figure 1B**, the plasma HbA1c level in the VDI group was remarkable higher than that of the normal VD group [(9.523 ± 2.35) % V.S. (7.85 ± 1.82) %, *t* = 4.60, *P*< 0.01). Results of Pearson test shows a significantly negative correlation between the plasma levels of HbA1c and 25(OH)D (*r* = -0.39, *P*< 0.01, **Figure 1C**).

### Glycolipid metabolic and inflammatory parameters in T2DM patients with normal VD or VDI and their relations with the plasma 25(OH)D level

**Figure 2:**
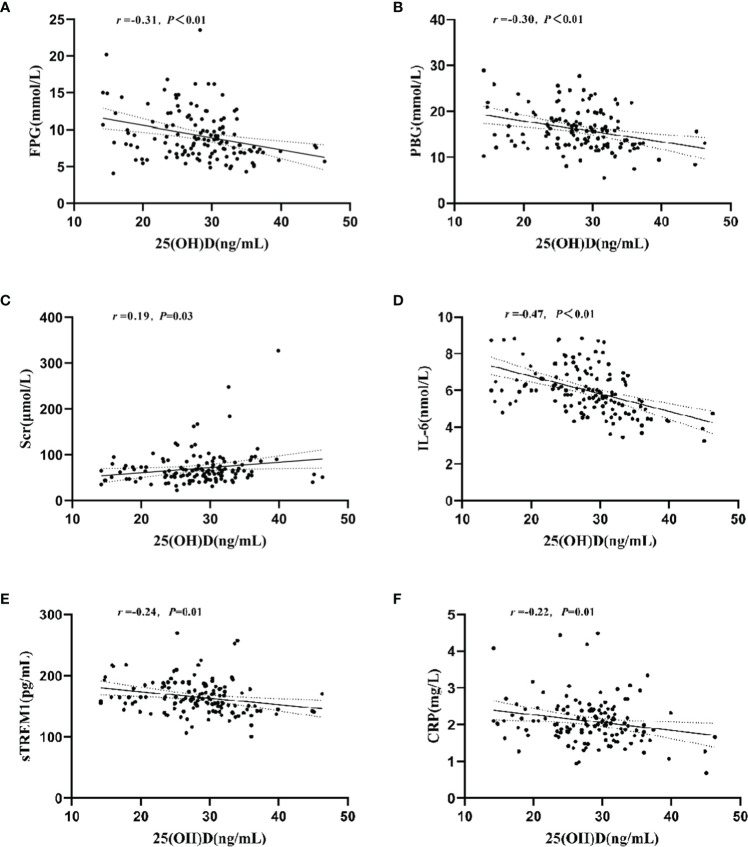
Relationship between plasma 25(OH)D level and plasma concentrations of FPG, PBG, Ser, IL-6, sTREM1, and CRP. **(A)** Correlation between plasma 25(OH)D and FPG levels; **(B)** Correlation between plasma 25(OH)D and PBG levels; **(C)** Correlation between plasma 25(OH)D and Scr levels; **(D)** Correlation between plasma 25(OH)D and IL-6 levels; **(E)** Correlation between plasma 25(OH)D and Strem1 levels; **(F)** Correlation between plasma 25(OH)D and CRP levels.

**Table 2** shows the biochemical indicators of glycolipid metabolism and inflammatory cytokines in T2DM patients with different plasma 25(OH)D level. Compared with that of the normal VD group, the plasma concentration of FPG (*t* = 3.01, *P<* 0.01), PBG (*t* = 2.75, *P<* 0.01), IL-6 (*t* = 4.64, *P<* 0.01), and sTREM1 (*t* = 2.08, *P* = 0.04) were significantly increased in T2DM patients with VDI group. However, there were no significant differences in other biochemical indicators between the two groups. Results of the correlation analysis showed that the plasma 25(OH)D levels were negatively associated with the levels of FPG (*r* = -0.31, *P<* 0.01, **Figure 2A**), PBG (*r* = -0.30, *P<* 0.01, **Figure 2B**), but positively correlated with SCR (*r* = 0.19, *P* = 0.03, **Figure 2C**). Moreover, the plasma 25(OH)D level was negatively correlated with the inflammatory molecules IL-6 (*r* = -0.47, *P*<0.01, **Figure 2D**), sTREM1 (*r* = -0.24, *P<* 0.01, **Figure 2E**), and CRP (*r* = -0.22, *P* = 0.01, **Figure 2F**).

**Table 2:** The biochemical indicators of glycolipid metabolism and inflammatory cytokines in T2DM patients with different plasma 25(OH)D levels.

Biochemical criterion	VDI group	Normal VD group	*t* value	*P* value
**TC (mmol/L)**	4.90 ± 0.17	4.79 ± 0.20	0.43	0.67
**TG (mmol/L)**	1.96 ± 0.21	1.91 ± 0.31	0.15	0.88
**HDL-C (mmol/L)**	0.97 ± 0.03	0.98 ± 0.04	0.25	0.8
**LDL-C (mmol/L)**	3.03 ± 0.13	3.00 ± 0.15	0.14	0.89
**ALT (U/L)**	24.61 ± 2.06	24.50 ± 2.77	0.03	0.98
**AST (U/L)**	20.42 ± 1.31	19.89 ± 1.32	0.29	0.78
**TBIL (μmol/L)**	16.64 ± 0.85	15.74 ± 0.97	0.7	0.49
**DBIL (μmol/L)**	4.32 ± 0.30	4.15 ± 0.30	0.39	0.7
**IBIL (μmol/L)**	12.51 ± 0.65	11.74 ± 0.79	0.75	0.45
**FPG (mmol/L)**	10.05 ± 0.44	8.36 ± 0.34	3.01	< 0.01
**PBG (mmol/L)**	17.08 ± 0.53	14.94 ± 0.51	2.75	0.01
**Scr (μmol/L)**	66.58 ± 3.34	74.14 ± 5.89	1.15	0.26
**BUN (mmol/L)**	5.44 ± 0.24	5.89 ± 0.33	1.09	0.28
**CRP (mg/L)**	2.15 ± 0.76	2.04 ± 0.08	1.09	0.28
**IL-6 (nmol/L)**	6.46 ± 0.14	5.49 ± 0.16	4.64	< 0.01
**sTREM1 (pg/mL)**	27.49 ± 3.29	26.96 ± 3.42	2.08	0.04
**sTREM2 (pg/mL)**	127.87 ± 2.02	127.97 ± 2.24	0.04	0.97

### CES-D sum scores in T2DM patients with normal VD or VD land the relation with plasma 25(OH)D level

**Figure 3:**
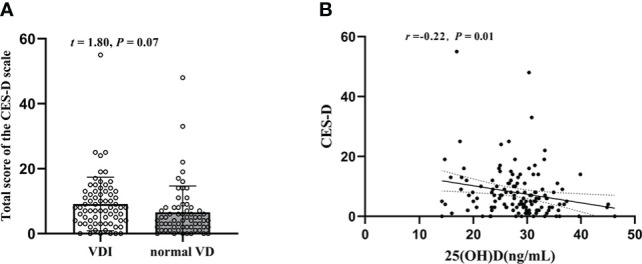
Changes of the CES-D sum scores in T2DM patients and their relation with plasma 25(OH)D level. **(A)** The CES-D sum scores in 25(OH)D<29(ng/mL) and HbA1c≥29(ng/mL); **(B)** Correlation between plasma 25(OH)D level and CES-D sum scores.

As shown in **Figure 3A**, compared with that of the normal VD group, there was a slight but not significant increase of the CES-D sum scores in T2DM patients with VDI group (*t* = 1.80, *P* = 0.07). The mean values of CES-D sum scores in the two groups were 9.13 ± 8.23 and 6.56 ± 8.09, respectively. However, there was a significantly negative correlation between the CES-D and 25(OH)D (*r* = -0.22, *P* = 0.01, **Figure 3B**).

### PSQI sum scores in T2DM patients with normal VD or VDI, and the relation with plasma 25(OH)D level

**Figure 4:**
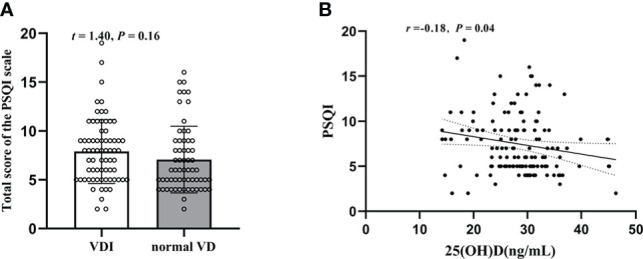
Changes of the PSQI sum scores in T2DM patients and their relation with plasma 25(OH)D level. **(A)** The PSQI sum scores in 25(OH)D<29(ng/mL) and HbA1c≥29(ng/mL); **(B)** Correlation between plasma 25(OH)D level and PSQI sum scores.

As shown in **Figure 4A** regarding PSQI, there was no significant difference between the two groups (*t* = 1.40, *P* = 0.16), with a PSQI sum score of 7.08 ± 3.42 in T2DM patients with normal VD level and 7.90 ± 3.28 in T2DM patients with VDI. Nonetheless, the plasma 25(OH)D level is negatively correlated with the PSQI sum scores (*r* = -0.18, *P* = 0.04, **Figure 4B**).

### BRIEF-A scores in T2DM patients with normal VD or VDI, and the relations with plasma 25(OH)D level, inflammatory cytokines and biochemical indicators

**Figure 5:**
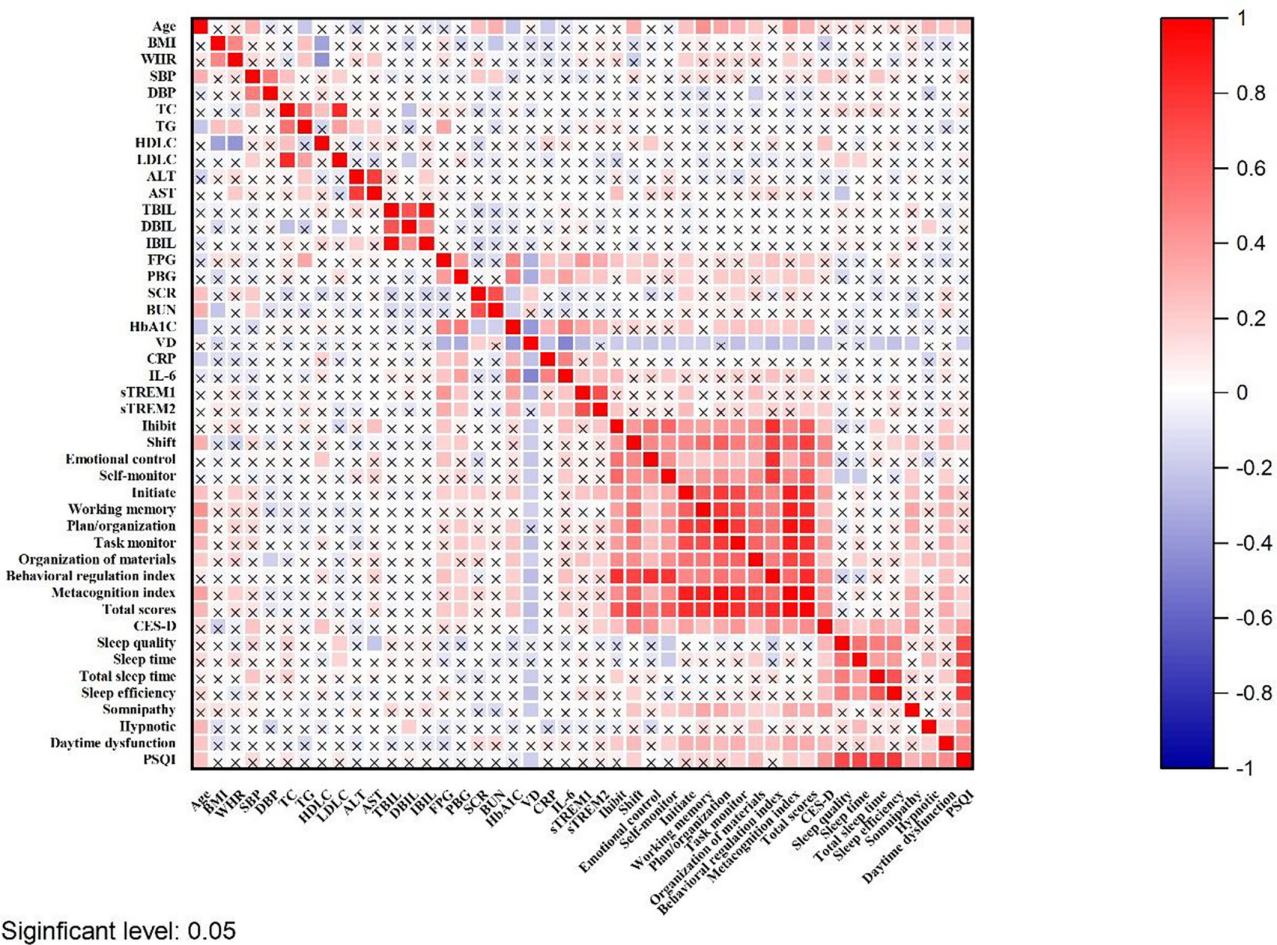
The correlation between BRIEF-A and 25(OH)D level, inflammatory cytokines and biochemical indicators in T2DM patients. The shade of the square color represents the size of the correlation; The symbol X means not statistically significant.

As shown in **Table 3**, there were significant differences in the scores of BRIEF-A between VDI and normal VD group. The BRIEF-A scores with VDI were significantly higher than the normal, including all subscales and two sub-entries (inhibit: *t* = 2.60, *P* = 0.01; shift: *t* = 2.60, *P* = 0.01; emotional control: *t* = 2.77, *P*<0.01; self-monitor: *t* = 2.43, *P* = 0.02; initiate: *t* = 3.20, *P*<0.01; working memory: *t* = 2.31, *P* = 0.02; plan/organization: *t* = 2.88, *P*<0.01; task monitor: *t* = 2.98, *P*<0.01; organization of materials: *t* = 3.37, *P*<0.01; behavioral regulation index: *t* = 3.40, *P*<0.01; metacognition index: *t* = 3.39, *P*<0.01; total scores: *t* = 3.81, *P*<0.01). Meanwhile, as shown in **Figure 5**, the plasma 25(OH)D level was negatively correlated with the BRIEF-A total scores, BRI, and MI scores. Furthermore, BRIEF-A had positive correlations with glycolipid metabolic parameters (FPG, PBG), inflammatory cytokine (IL-6, sTREM1), CES-D and PSQI sum scores.

**Table 3:** Differences of BRIEF-A scores in T2DM patients with different 25(OH)D levels.

BRIEF-A	VDI group	Normal VD group	*t* value	*P* value
**Inhibit**	53.70 ± 1.27	49.50 ± 0.99	2.60	0.01
**Shift**	57.76 ± 1.18	53.23 ± 1.30	2.60	0.01
**Emotional control**	56.24 ± 1.49	51.13 ± 1.09	2.77	< 0.01
**Self-monitor**	52.71 ± 1.30	48.65 ± 1.05	2.43	0.02
**Initiate**	62.39 ± 1.83	54.60 ± 1.55	3.20	< 0.01
**Working memory**	67.31 ± 1.79	61.45 ± 1.78	2.31	0.02
**Plan/organization**	62.51 ± 1.79	55.63 ± 1.55	2.88	< 0.01
**Task monitor**	56.44 ± 1.34	51.08 ± 1.18	2.98	< 0.01
**Organization of materials**	48.91 ± 1.05	44.16 ± 0.92	3.37	< 0.01
**Behavioral regulation index**	220.41 ± 4.03	202.50 ± 3.41	3.40	< 0.01
**Metacognition index**	297.57 ± 6.76	266.92 ± 5.85	3.39	< 0.01
**Total scores**	517.99 ± 9.40	469.42 ± 8.42	3.81	< 0.01

The effect of age on scores was ruled out.

### ROC curve of plasma 25(OH)D level in distinguishing high total scores of BRIEF-A (scores > 50th percentile) from low total scores of BRIEF-A (scores ≤ 50th percentile) in T2DM patients

**Figure 6:**
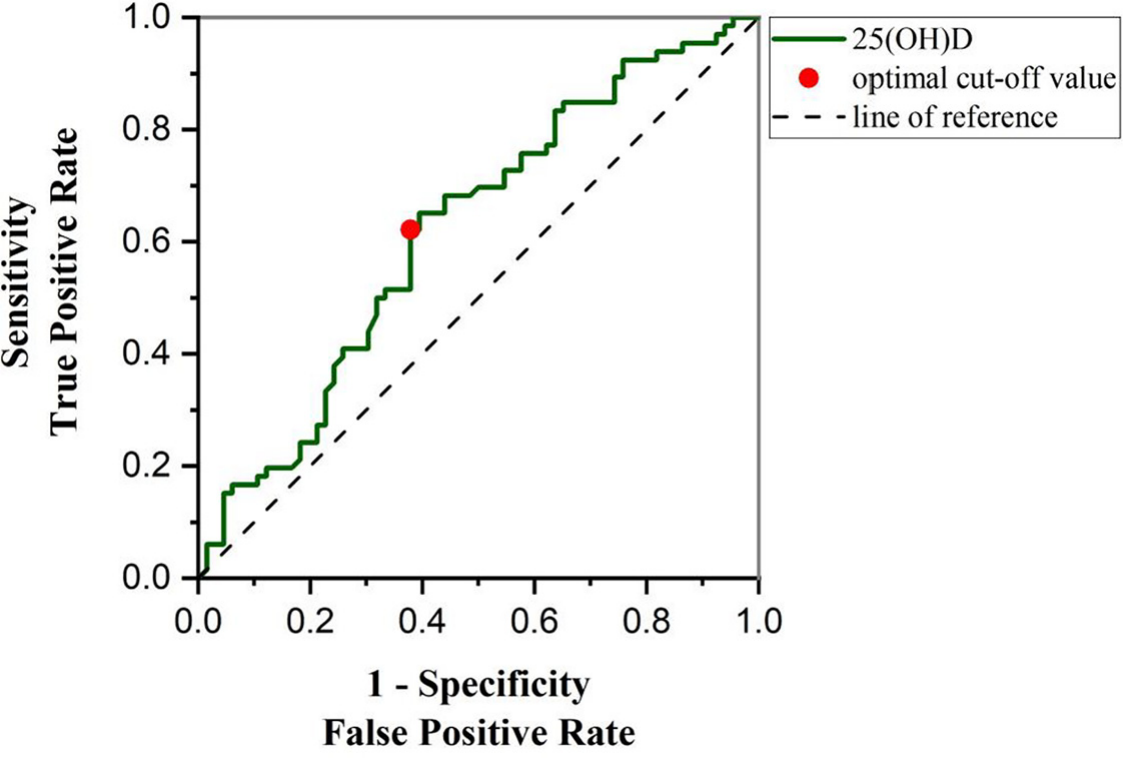
ROC curve was used to analyze the diagnostic significance of 25(OH)D level to distinguish high total scores of BRIEF-A (scores > 50th percentile) and low total scores of BRIEF-A (scores ≤ 50th percentile). 25(OH)D concentration was 28.48 ng/mL, the sensitivity and specificity were 62.12% and 62.12%, and the AUC value was 0.62(95% CI: 0.5252-0.7166), which could be used as the best critical point for differentiating T2DM patients with high total scores of BRIEF-A and low total scores of BRIEF-A. AUC, area under the curve.

ROC curve analysis was used to determine the significance of BRIEF-A level in the T2DM patients by 25(OH)D. We used the total scores of BRIEF-A for a rough analysis, and used the 50th percentile of the total score as the high and low boundary. As shown in **Figure 6**, 25(OH)D has specificity and sensitivity in differentiating between the high and low scores of BRIEF-A, suggesting that 25(OH)D has potential diagnostic value for judging BRIEF-A in clinical practice. The AUC area under the curve was 0.62 (95% confidence interval 0.525-0.717), and the 25(OH)D critical value was 30.5. The sensitivity and specificity were 62.12% and 62.12%, respectively.

## Discussion

In this study, we investigated the plasma 25(OH)D level in T2DM patients and its correlation with the glucolipid metabolic dysfunction, inflammatory activation, depressive symptoms, sleep quality and cognitive impairment. The results revealed that plasma 25(OH)D levels were statistically significantly lower in T2DM patients with HbA1c ≥ 7% compared to the HbA1c< 7% group. Taken the clinical diagnosis criteria of VDI, our results showed that T2DM patients with VDI had a higher level of HbA1c, inflammatory cytokine, and BRIEF-A scores than patients with normal VD level. Results of the correlation analysis showed negative correlations between 25(OH)D and glycolipid metabolic parameters (HbA1c, FPG, PBG), depressive symptomatology (assessed by CES-D sum score), sleep disorders (assessed by PSQI sum sores), and three inflammatory markers (CRP, IL-6, sTREM1). And 25(OH)D level was positively associated with the biochemical indicator Scr. Moreover, results of the ROC curve showed a predictive role of 25(OH)D in discriminating T2DM patients with high scales of BRIEF-A (scores > 50th percentile) from low scores (scores< 50th percentile). These results indicated that VDI is likely be a risk and predicative factor for cognitive impairments in T2DM patients, and involved with the inflammatory response and glucolipid metabolism.

It has been reported that 25(OH)D status are involved with the process of T2DM and its complications (36), with a regulatory role on glucolipid metabolism and insulin resistance (37, 38). Moreover, in patients with a deficiency of 25(OH)D, there was a higher HbA1c as well as disorders of blood lipids (39). The results of our study showed that T2DM patients with VDI has a higher HbA1c, which has been a clinical indicator of predicting the risk of diabetic complications (40). Moreover, apart from the negative correlation between plasma 25(OH)D level and HbA1c, our results also showed that the plasma 25(OH)D level was also negatively related to FPG and PBG, but positively correlated with Scr. Together with the findings that T2DM were more susceptible to VDI (41). These above suggest that patients should be taken to the harmful effect of VDI in the pathogenesis or T2DM.

Excessive inflammation and immune activation have long been taken as etiological and concomitant conditions of T2DM (42). It has been reported that hyperglycemia and metabolic disorders could trigger the inflammatory response *via* incur an imbalance between the oxidizing species, oxidative stress and cellular death (43). 25(OH)D is essential in maintaining the normal immune system and modulating inflammation of the innate immune function (44). Besides, 25(OH)D may regulate inflammatory gene expression by suppressing endoplasmic reticulum stress, oxidative stress (45). Moreover, supplementation with 25(OH)D can improve metabolic disorders and alleviate inflammatory response of T2DM patients *via* reducing a number of inflammatory factors such as IL-6, CRP, and TNF-α levels (46, 47). Consistently, in the present study, our results showed that the plasma levels of IL-6 and sTREM1 were remarkable higher in T2DM patients with VDI than that the normal VD, with a negative correlation between plasma 25(OH)D concentrations and IL-6, sTEREM1, or CRP. These results suggested that VDI might play a detrimental role in the hyper-inflammatory response of T2DM patients.

Cognitive impairment is a common comorbidity or complication of T2DM (2). Besides poor glycemic control, the reported risk factors include hyperinsulinemia, increased oxidative stress (48) depression, and sleep disorders (49, 50). In line with these findings, in the present study, our results showed that the BRIEF-A scores were positively correlated with the FPG, PBG, IL-6, sTREM1, CES-D and PSQI sum scores, suggesting again a close relationship between the cognitive function and glycolipid metabolic parameters, inflammatory cytokines, depressive emotion, and sleep state. It has been reported that T2DM patients with cognitive impairment were at higher risk of low 25(OH)D level as compared to their healthy counterparts (51). Consistently, results of the present study demonstrated that compared with the normal VD group, the BRIEF-A scores of the VDI was higher, which indicated a worse cognitive competence of the VDI patients than that of the normal. In further, results of Pearson test showed that the plasma 25(OH)D level was negatively correlated with the BRIEF-A total scores, BRI, and MI scores. Also, 25(OH)D was negatively correlated with the risk factors for cognitive dysfunction, including inflammation, depressive emotion and sleep state. These results suggested that 25(OH)D played a crucial role in the cognitive dysfunction of T2DM patients. Although there has been demonstrated that low level of 25(OH)D was significantly associated with sleep disturbance and depression (52), and vitamin D supplements could improve the sleep score and quality of sleep disorders aging 20-50 (53), our results showed that there was no significant different between groups with regard to the depression and sleep status. Nevertheless, the indices of depression and sleep were slightly impaired in T2DM patients with VDI, with a significantly negative correlations between plasma 25(OH)D level and the CES-D or PSQI sum scores. It may suggest that 25(OH)D indeed has an effect on the depressive symptoms and sleep disturbances that accompany T2DM, but the extent of this is not known.

In this study, ROC curves were used to suggest the potential value of 25(OH)D in diagnosing cognitive function. The higher the sensitivity and the lower the 1-specificity in the ROC curve analysis, the more reliable an indicator is as a basis for diagnosis. We obtained a sensitivity and specificity of 62.12% and an area under the AUC curve of 0.62. These results suggest that 25(OH)D has a certain diagnostic basis for cognitive function in patients with T2DM. Nevertheless, 25(OH)D as a diagnostic criterion has a certain rate of misdiagnosis. Insufficient sample size is the main reason, which means lower power of test. Further higher accuracy might be obtained by expanding the sample size. In addition, other factors, such as age, may also have a potential impact. One study indicated that 25(OH)D deficiency was more closely associated with cognitive dysfunction in older patients (54). But the majority of our study participants were in middle age. These mean that we need to consider additional factors in the discussion of 25(OH)D as a basis for diagnosis, and further investigate its value while expanding the sample size.

There are several limitations in the present study. Firstly, the relatively small sample size limits the generalizability of the present results. Furthermore, although the changed 25(OH)D level and its correlation with some glucolipid, inflammation, or cognitive function was observed in T2DM patients, the detailed mechanism was not explored in the present study. Moreover, prospective studies with vitamin D supplements would have to warrant our findings in the future.

## Conclusion

In conclusion, our study indicated that VDI should be a detrimental factor in the pathogenesis of T2DM, involved with not only the glucose metabolism and inflammatory response, but also the cognitive function. Moreover, plasma 25(OH)D level might play a predictive role in discriminating T2DM patients with higher cognitive impairments.

## Data availability statement

The raw data supporting the conclusions of this article will be made available by the authors, without undue reservation.

## Ethics statement

The studies involving human participants were reviewed and approved by the Ethics Committee of Hefei Fourth People’s Hospital, Anhui Mental Health Center. The patients/participants provided their written informed consent to participate in this study. Written informed consent was obtained from the individual(s) for the publication of any potentially identifiable images or data included in this article.

## Author contributions

J-FG designed this study. H-MS, YY, X-RG conducted most of the experiments, analyzed the data and wrote the manuscript. J-FG conceived the study and revised the manuscript, H-MS analyzed the results and write text. YY screened and selected the patients, collected material. X-RG processed the samples, analysis results. And Y-DW, C-ZQ, M-DM, D-DX and Y-YX assisted in the experiments. All authors contributed to the article and approved the submitted version.
