# Supplementary material for: Potential role of 25(OH)D insufficiency in the dysfunction of glycolipid metabolism and cognitive impairment in patients with T2DM
Source: Front Endocrinol (Lausanne). 2022 Dec 23;13:1068199. doi: 10.3389/fendo.2022.1068199 (PMC9822724; doi:10.3389/fendo.2022.1068199)
Supplement: Supplementary file 1 [file DataSheet_1.pdf]

# Supplementary Figure

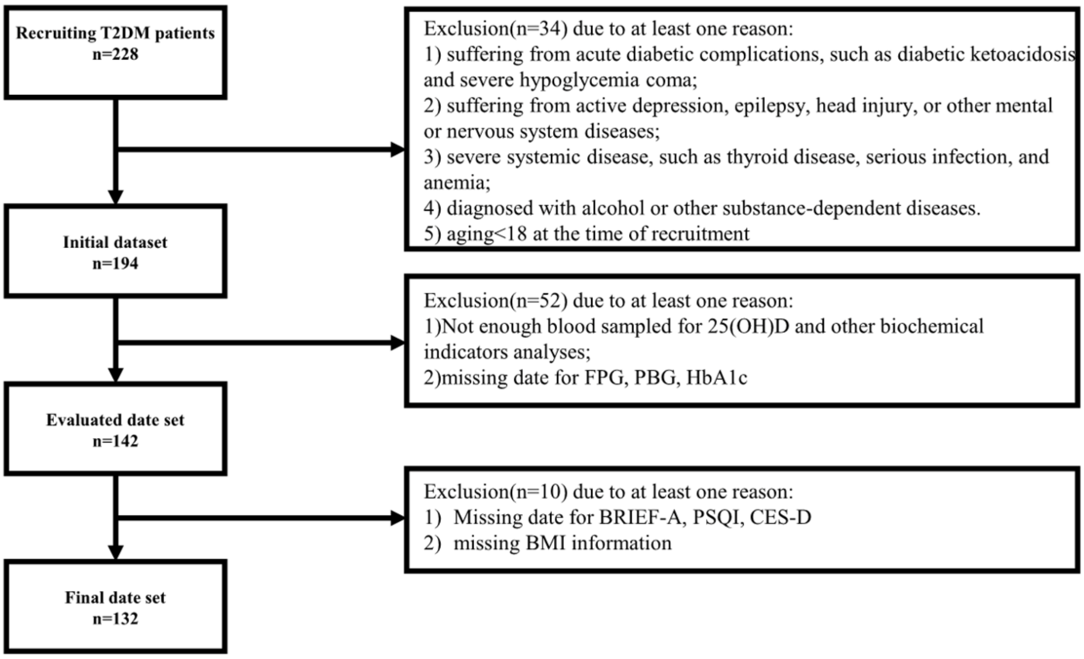


**Figure S1. Flow diagram of sample selection process.**

**Note:** FPG: fasting blood glucose, PBG: postprandial blood glucose, HbA1c: glycated hemoglobin, BRIEF-A: Behavior Rating Inventory of Executive Function–Adult Version, PSQI: Pittsburgh sleep quality index, CES-D: German version of the Centre for Epidemiological Studies Depression Scale, BMI: body mass index,
